# Prenatal Temperature Shocks Reduce Cooperation: Evidence from Public Goods Games in Uganda

**DOI:** 10.3389/fnbeh.2017.00249

**Published:** 2017-12-21

**Authors:** Jan Duchoslav

**Affiliations:** Development Economics Group, Wageningen University, Wageningen, Netherlands

**Keywords:** climate change, temperature shocks, public goods game, cooperation, fetal origins, Africa

## Abstract

Climate change has not only led to a sustained rise in mean global temperature over the past decades, but also increased the frequency of extreme weather events. This paper explores the effect of temperature shocks *in utero* on later-life taste for cooperation. Using historical climate data combined with data on child and adult behavior in public goods games, I show that abnormally high ambient temperatures during gestation are associated with decreased individual contributions to the public good in a statistically and economically significant way. A 1 standard deviation rise in mean ambient temperature during gestation is associated with a 10% point decrease in children's cooperation rate in a dichotomous public goods game, and the reduced taste for cooperation lasts into adulthood.

## 1. Introduction

**Figure 1:**
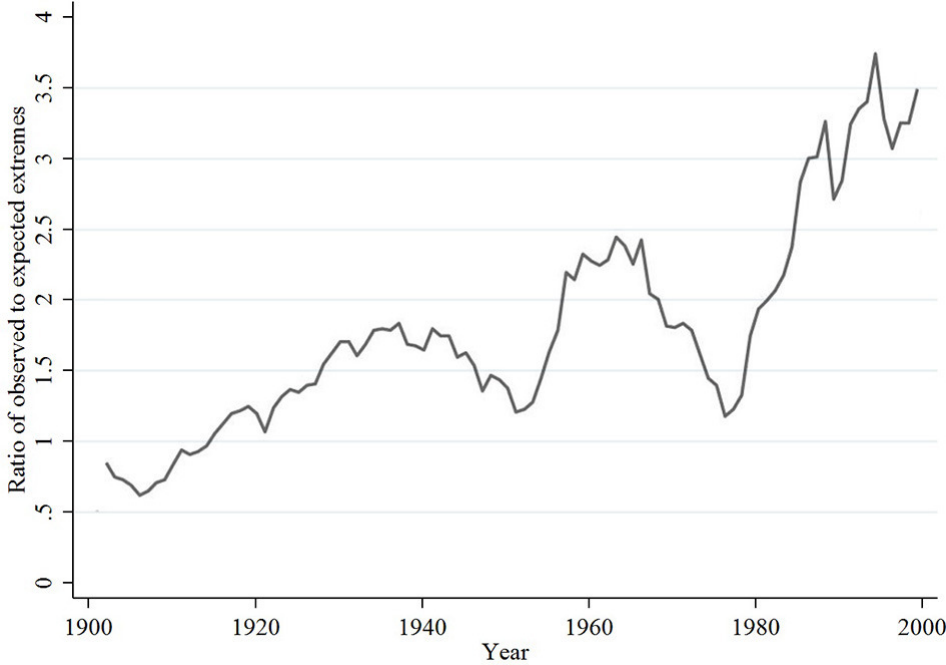
Extreme temperatures.

Climate scientists have reached solid consensus that global climate change is occurring over a decade ago (Oreskes, 2005). There has been a sustained rise of mean global temperature, and extreme temperatures have become increasingly common (see Figure 1, adapted from Coumou and Rahmstorf, 2012). The focus of scientific discourse on the topic has therefore shifted toward estimating the economic implications of future climate change as well as finding feasible, effective countermeasures and mitigation strategies (Dell et al., 2009). The severity of the former justifies the costs of the latter. Careful assessment of the damage function is thus of utmost importance.

Recent contributions to this literature have investigated the effects of immediate temperature on outcomes ranging from economic production (Dell et al., 2009; Burke et al., 2015) through the onset of conflict (Hsiang et al., 2013), to mortality rates (Barreca et al., 2016) and human reproductive behavior—with consequences for physical health and educational outcomes of the offspring (Wilde et al., 2017), and potentially for overall population growth (Barreca et al., 2015). Inspired by another growing body of literature—that on fetal origins, i.e., the impact of intrauterine conditions during gestation on later-life outcomes—I take a step back and consider behavioral implications of temperature shocks *in utero*. Using historical variation in ambient temperature as a natural experiment, and behavior in an incentivized public goods game as an outcome measure, I assess the impact of unusually high temperatures during gestation on later-life taste for cooperation—a preference essential to much economic production. I find that abnormally high ambient temperatures during gestation significantly reduce cooperativeness in children, and that this effect lasts into adulthood.

Stemming from an observation by the epidemiologist Barker (1990) that low birth-weight and premature birth are associated with coronary heart disease in later life, the fetal origins literature has grown considerably beyond the medical field into other domains including economics, psychology or management science. Conditions *in utero* and their proxies have now been linked to later life outcomes ranging from educational achievement (Bhutta et al., 2002; Almond, 2006) through trading ability (Coates et al., 2009) to sexual identity (Csathó et al., 2003). Using the 1918 influenza pandemic as a natural experiment, Almond (2006) finds that mother's illness during pregnancy reduces the educational attainment and income of the offspring. Other natural experiments make use of the Ramadan (Almond and Mazumder, 2011) and the Nazi invasion of the Netherlands (van Os and Selten, 1998) to show that fasting and stress (respectively) during pregnancy increase the chance of mental disability in the offspring. In a similar fashion, Banerjee et al. (2010) use the case of the advancing phylloxera infestation of French vineyards to show that negative income shocks during gestation reduce adult height—a marker of overall health.

In another strand of the fetal origins literature, various markers of conditions *in utero* such as preterm birth, the ratio between the lengths of the index and ring fingers (2D:4D), and especially weight at birth are linked to later-life outcomes. As a direct consequence of intrauterine growth retardation, preterm birth, or both, low birth weight is a telltale sign of adverse conditions *in utero*. The exact nature of the physiological processes that lead to low weight at birth (often collectively referred to as intrauterine programming) are still subject to vigorous scientific debate. There is however growing consensus that they may involve hormonal imbalances in early pregnancy, decreased fetal nutritional intake in late pregnancy (whether due directly to low maternal nutritional intake or to suboptimal placental size, blood flow or function), and low fetal oxygen supply throughout gestation. These can in turn be triggered by conditions as diverse as maternal malnutrition, stress, disease, substance abuse, and environmental exposure (such as to high altitude or ambient temperature) (Fowden et al., 2006). As a general marker of unfavorable intrauterine conditions, low birth weight has been linked to various later-life outcomes ranging from cardiovascular disease (Barker, 1990) to lower income (Black et al., 2007; Bharadwaj et al., 2017), behavioral problems (Hille et al., 2007), and reduced cognitive abilities (Hack et al., 2005; Figlio et al., 2014), which in turn reduce the taste for cooperation (Moore et al., 1998; Zhang et al., 2015)^1^.

Considering the abundant evidence that ambient temperature during gestation is one of the factors affecting birth weight (Wells and Cole, 2002; Lawlor et al., 2005; Deschênes et al., 2009) and preterm birth (Lajinian et al., 1997; Yackerson et al., 2008; Flouris et al., 2009), its effects on later-life outcomes in general and social preferences in particular have received surprisingly little attention. To be sure, much of this non-experimental strand of fetal origins literature consists of comparative cohort studies without sufficient controls for socioeconomic and behavioral confounders (Black et al., 2007; Dell et al., 2009; Deschênes et al., 2009; Zhang et al., 2015 being noteworthy exceptions), and is therefore prone to suffer from omitted variable bias. Taken as a whole, this body of literature nonetheless points toward a link between ambient temperature during gestation and later-life outcomes. The methodologically well-executed study by Deschênes et al. (2009) (as well as that by Lawlor et al., 2005) further suggests that it is relative—rather than absolute—temperature shocks that matter in this respect^2^.

To my knowledge, the hitherto only study to look at the effect of *in utero* temperature shocks on later-life outcomes links temperature during gestation to depression in adulthood (Adhvaryu et al., 2015). The present paper fills in part of the remaining gap by studying the effects of ambient temperature during gestation on the taste for cooperation. I describe the experimental design and my empirical strategy in section 2, present the results in section 3, and conclude in section 4.

## 2. Experimental design and data

Employing new data from behavioral games, anthropometric measurements and an extensive socioeconomic survey conducted in Uganda, I exploit the quasi-experimental variation in weather to gauge the impact of prenatal temperature shocks on later-life cooperation.

I use several distinct datasets in my analysis^3^. Temperature data come from Willmott and Matsuura's (2015) gridded monthly time series interpolated from weather station observations. I combine the temperature values with my main and secondary self-collected datasets. The main set contains data from a survey of primary school pupils from Northern Uganda, and also includes their choices in a one-shot dichotomous public goods game, as well as their anthropometric measurements. The secondary set contains data from a household survey from Southern Uganda, and records of the behavior of the representatives of these households in a standard public goods game.

### 2.1. Temperature

I construct my measure of ambient air temperature during gestation using Willmott and Matsuura's (2015) historical time series—one of two publicly available datasets with values spatially intrapolated from terrestrial weather station measurements. Although the alternative dataset produced by Harris et al. (2014) is generally more popular, that of Willmott and Matsuura is better suited for my purposes as it uses a much denser set of weather stations in East Africa (as well as globally)^4^. The dataset contains a single temperature value for each historical month and spatial grid cell of 0.5 × 0.5° (roughly 55 × 55km in Uganda).

My behavioral and survey data come from two clusters of locations—one in Northern Uganda, spanning four neighboring grid cells (2.5–3.5°N, 32.5–33.5°E), and one from a single grid cell in Southern Uganda (0.5–1.0°S, 30.0–30.5°E). Since the differences between the values in the four neighboring northern cells in any given month are minimal, I use their mean values for all observations in the northern cluster, obtaining a single monthly temperature value for each of the two location clusters. Temperature variation within each cluster of location thus stems from temporal, rather than geographical differences. To obtain the value of ambient temperature during the gestation of a respondent in the northern cluster, I average the temperature in the northern location during the month of his or her birth and in the preceding 8 months. The values for respondents from the southern locations are constructed analogously. For an individual born in November, for example, I average the monthly values from March until November.

**Figure 2:**
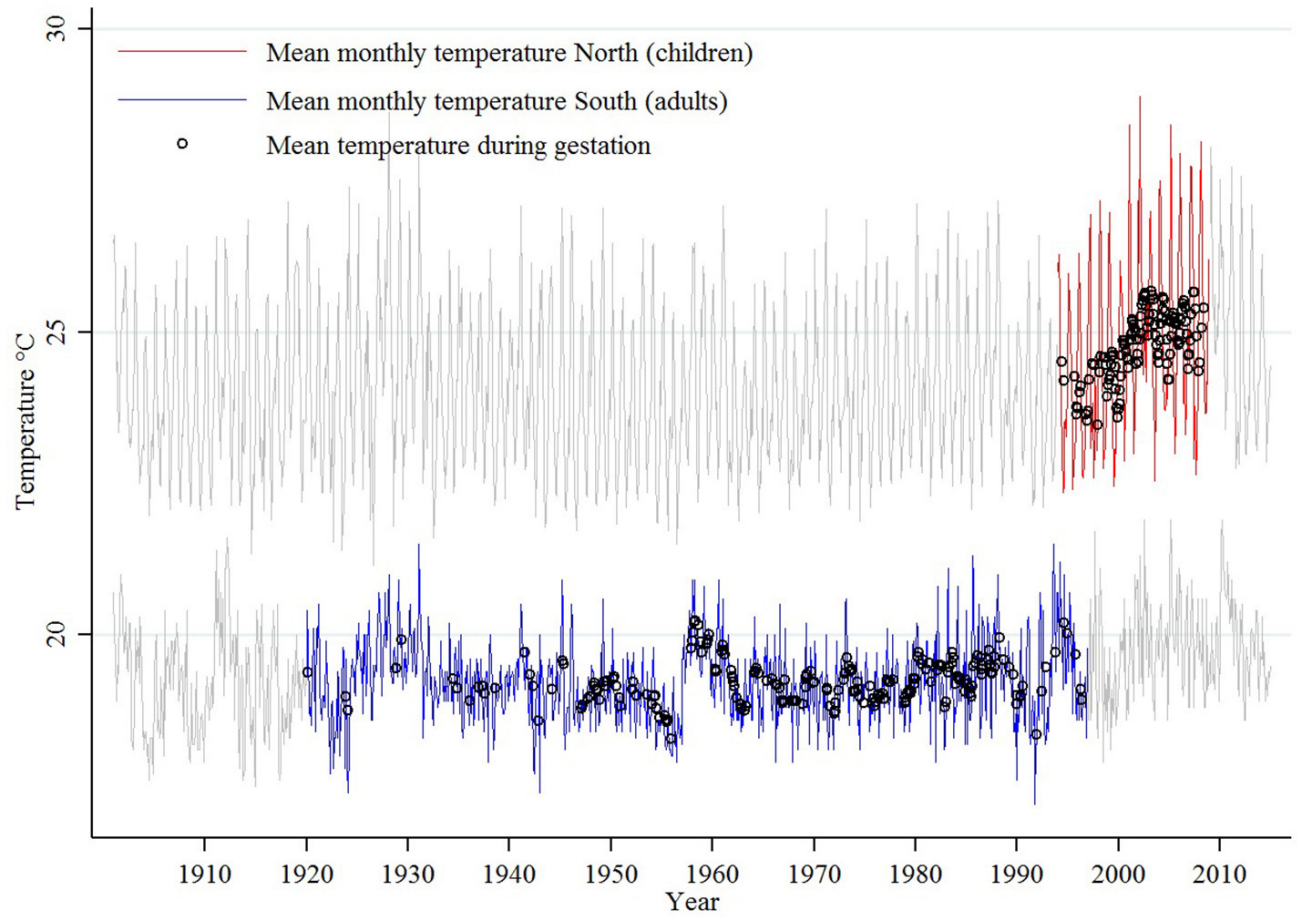
Temperature trends.

The historical monthly means are plotted in Figure 2, where the gray curves represent the monthly temperature means in the two clusters, the red curve represents the temperatures in the months in which the respondents in the northern cluster (main sample) were gestating, and the blue curve denotes the temperatures in the months in which the respondents in the southern cluster (secondary sample) were gestating. The mean values of ambient air temperature during the gestation (9 months) of individual respondents are denoted by black circles.

Mean temperatures of 9-month-long gestational periods have, by construction, a much smaller variance than monthly mean temperatures (as Figure 2 illustrates). Similarly, the variance of monthly means is smaller than that of daily means. Basing my analysis on overall mean values therefore somewhat reduces its sensitivity. Using more detailed temperature data such as a set of 9 monthly values for each individual would not, however, correspond to the level of precision with which I can determine the dates of conception—and thus the periods of gestation—of the respondents. By its nature, I can only infer an individual's probable date of conception from their date of birth. The possibility of premature and late births introduces in such inference a level of uncertainty which is only aggravated by the fact that I only know the month (rather than the exact date) of birth of my respondents. The margin of error associated with these imprecisions can easily be more than a month. In extreme cases, there would thus be no overlap between actual and assumed values of temperatures in any given month of gestation. Using instead the mean value over the whole assumed period of gestation largely reduces the effect of such inaccuracies.

### 2.2. Main sample

My main sample consists of 531 children and their caregivers from Pader district in Northern Uganda. The children come from 42 primary schools visited in June and July 2014. In each school, 16 pupils were randomly selected from a list of those enrolled at the beginning of the year^5^.

I measure children's and caregivers' willingness to cooperate by involving them in a one-shot dichotomous public goods game similar to those in Cárdenas et al. (2009) and Barr et al. (2014). In each school, children were randomly assigned to groups of 8, but were not told which other 7 children (of the 15 participating in that school) belonged to their group. Each child then anonymously selected either a “private card” or a “group card”^6^. By choosing the private card, the respondent allotted 4 candies to himself, but none to the other unknown members of the group. By selecting the group card, the respondent instead ensured 1 candy for each of the 8 group members, including himself (see Appendix C in Supplementary Materials for a reproduction of the two cards). In this set up, total welfare is maximized when all 8 game participants opt for the group card, such that they each receive 8 candies. A sole free rider selecting the private card would receive 11 candies, but in the Nash equilibrium, everyone selects the private card and ends up with only 4 candies each.

Caregivers played a similar public goods game, but made their decisions in the isolated environment of their home, unaware of the identity of the other 8 participants with whom they were grouped. If they chose the private card, they received 4,000 UGX (roughly 1.5 USD at the time). Choosing the group card instead meant an allocation of 1,000 UGX to each anonymous member of the group, including themselves. In the Nash equilibrium, each participant thus received 4,000 UGX, total welfare was maximized at a return of 8,000 UGX for each group member, and a sole free rider would earn 11,000 UGX. 26% of the children chose the cooperative option, while the cooperation rate among their caregivers was 34%.

The descriptive statistics for the children are presented in Table 1A. The mean ambient temperature faced by the mothers of the children in my sample during their pregnancy was 24.9°C. The children are on average 10 years old, and girls and boys are equally represented. The height of the children in the sample is practically identical to the mean for their age, but their body mass is 1.39 standard deviation below the mean for their age (de Onis et al., 2007)^7^. This suggests that some may have been nutritionally deprived in their early life, which could confound my results (I address this issue below). Children's cognitive ability was measured through standard Raven's progressive matrices (Kaplan and Saccuzzo, 2012). It is an intelligence quotient (IQ) adjusted for age and scaled relative to the sample (with a mean at 100 and a standard deviation of 15). I further proxy for child prenatal stress by the second-to-fourth (2D:4D) digit ratio—a marker of hormonal exposure *in utero*^8^.

**Table 1:** Descriptive statistics (main sample).

	**Mean**	**Std. dev**.	**Min**.	**Max**.	***N***
**(A) CHILDREN**					
Temperature	24.915	0.505	23.464	25.681	531
Cooperation	0.256	0.437	0	1	531
Female	0.493	0.5	0	1	531
Age in years	10.478	2.731	6	20	531
Height-for-age	−0.025	1.005	−3.391	3.571	516
BMI-for-age	−1.387	0.935	−3.99	2.26	517
IQ-for-age	100.619	14.815	73.993	148.458	488
Prenatal stress	0	1	−3.273	5.028	215
Postnatal conflict exposure	0	1	−4.971	2.885	477
Precipitation	3.507	0.559	2.401	5.124	531
Consumer prices	−2.113	2.163	−5.954	4.937	531
**(B) CAREGIVERS**					
Age in years	40.588	9.738	20	94	522
Age at birth of child	30.207	9.545	10	85	522
Female	0.58	0.494	0	1	531
Acholi	0.976	0.155	0	1	531
Christian	0.998	0.043	0	1	531
Years of education	3.84	3.431	0	18	531
Married	0.772	0.42	0	1	531
Functional literacy	0.234	0.423	0	1	531
Conflict exposure	0	1	−3.035	1.688	528
Household size	7.945	2.491	2	20	531
Wealth	0	1	−1.54	3.068	531
Risk averseion	0.516	0.5	0	1	531
Cooperation	0.335	0.473	0	1	531

**Figure 3:**
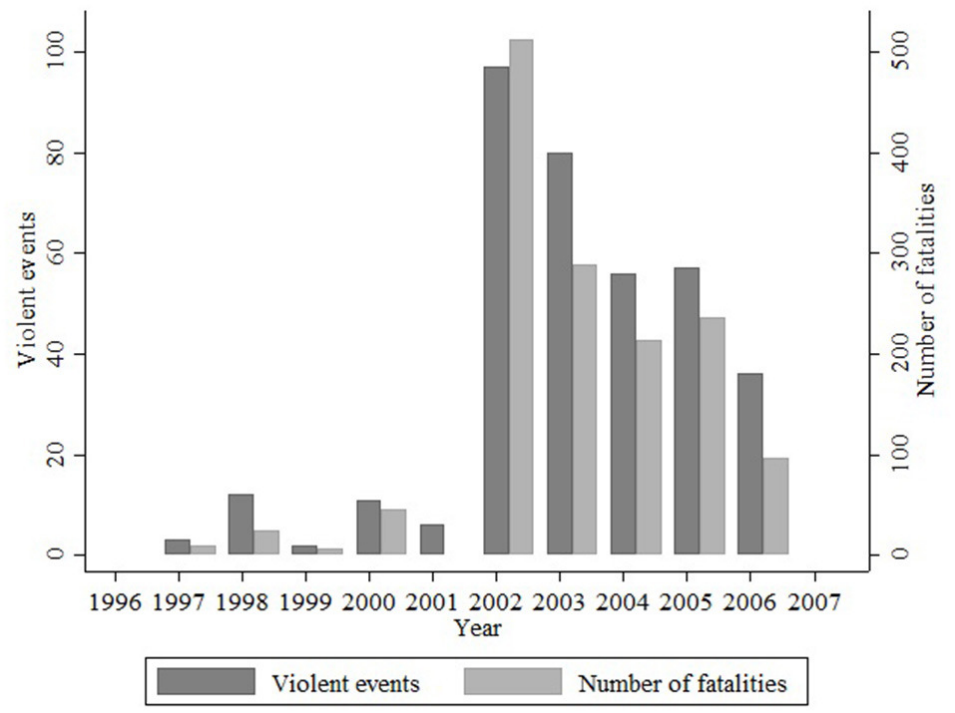
Civilian targeting.

Child postnatal conflict exposure—a potentially important confounding factor considering that most children in the sample were born during a period of civil war in Northern Uganda—is a composite measure derived from the exposure of the caregiver and the child's year of birth. Given their young age at the time of the conflict, children were not asked any war-related questions. Instead, I use caregiver responses to an adapted version of the War Trauma Questionnaire (Macksoud, 1992; Papageorgiou et al., 2000)^9^. It consists of 23 yes–no questions about various violent events witnessed by the caregiver, from which I construct a conflict exposure index using the number of positive responses as a measure of exposure (Bellows and Miguel, 2009) and normalizing it for the sample. To proxy the child's postnatal conflict exposure I weight the caregiver's conflict exposure index by the portion of violence their child could have potentially witnessed after birth. To obtain the weights, I divide the number of civilian fatalities that occurred in Pader district following the child's birth by the total number of civilian facilities recorded in the district throughout the length of the conflict (Figure 3)^10^. For example, a child born in December 2003—by which time 62% of reported fatalities took place—whose caregiver's conflict exposure is 87% is likely to have witnessed 38% of the violence that the caregiver was exposed to. For my purposes, the child's conflict exposure index would therefore be 33% ((1−0.62) × 0.87 = 0.33).

Finally, precipitation and consumer prices during gestation are constructed analogously to the temperature variable.

To account for further environmental and genetic effects on preferences, I also interviewed each child's main caregiver—the adult household member with whom the child spends most time. The descriptive statistics for the caregivers are presented in Table 1B. About half of the caregivers in my sample chose to cooperate in the public goods game. Caregivers are on average 41 years of age^11^, 58% are female. Additionally, I collected information about their education level and risk preferences. All caregivers were exposed to at least some kind of conflict-related violence, though the level of exposure varies greatly^12^. Almost all respondents are Christian and belong to the Acholi ethnic group. A typical household is composed of 8 people. I also collected information about their relative asset wealth (Sahn and Stifel, 2003).

In my setting, information about the current main caregivers can only serve as a proxy for environmental and genetic influences to which the children have been subjected throughout their lives. Of the 531 caregivers in my sample, only 265 are biological mothers of the children, while 206 are their biological fathers. The remaining 60 were grandparents, uncles or aunts, other relatives, and siblings (in descending order of prevalence). One caregiver was not related to the child at all. Nonetheless, the average caregiver in my sample had been taking care of the child for 82% of the child's life, making the information about the caregivers a strong proxy for the environment surrounding the children.

### 2.3. Secondary sample

My main dataset contains rich information about the children and their environment, but suffers from two important shortcomings. The first is its conflict setting. If temperature shocks invite conflict (O'Loughlin et al., 2012; Hsiang et al., 2013), then the physiological effects of ambient temperature during gestation would be hard to disentangle from the effects of temperature-induced conflict. Second, it does not allow me to repeat the analysis using the caregiver's behavior and temperature during their gestation, because I only know the caregivers' year of birth. This means that I cannot tell whether the behavioral effects that temperature shocks *in utero* have on children last into adulthood. To address these concerns, I turn to a second sample of 257 adults from Sheema district in Southern Uganda, which was untouched by the conflict in the north.

In July and August 2014 I visited 45 villages in the district, and surveyed a random sample of 10 households per village selected from a census. A randomly selected adult representative of each surveyed household was invited to participate in an incentivized public goods game^13^.

The game was played in groups of 5 participants who could anonymously decide to contribute between 0 and 5 tokens (worth 1,000 UGX or 0.38 USD each) to a common pot, keeping the rest for themselves. Shared funds were doubled and redistributed equally (after rounding). After an initial practice round, 5 rounds of the game were played with each group, though the participants did not know beforehand how many rounds the game would last^14^. One round was selected at random for payment.

In this design, total welfare is maximized when all participants contribute their entire endowment of 5 tokens to the common pot, receiving 10 each in return. Nevertheless, free riders could receive up to 13 tokens, and the Nash equilibrium is reached with all players keeping their 5 tokens. On average, participants contributed 3.44 tokens to the public good. The descriptive statistics for the game participants are presented in Table 2. The mean ambient temperature faced by the mothers of the adults in my sample during their gestation was 19.2°C. The participants are on average 42 years old, a third are female, and 84% are married. On average, they fell just short of completing primary education, and half are functionally literate. Nearly the whole sample is ethnically Ankole and Christian by religion.

**Table 2:** Descriptive statistics (secondary sample).

	**Mean**	**Std. dev**.	**Min**.	**Max**.	***N***
Temperature	19.222	0.347	18.278	20.233	257
Contribution	3.437	1.174	0	5	257
Age in years	42.004	15.711	16	92	257
Female	0.335	0.473	0	1	257
Years of education	6.743	3.866	0	17	257
Married	0.844	0.363	0	1	257
Munyankole	0.977	0.151	0	1	257
Christian	0.996	0.062	0	1	257
Functional literacy	0.498	0.501	0	1	257
Wealth	0	1	−1.212	5.515	257

## 3. Analysis and results

### 3.1. Main finding

I hypothesize that exposure to high ambient temperatures during an individual's gestation may impact his or her later-life preference for cooperation. Combining the findings of Wells and Cole (2002), Lawlor et al. (2005), and Deschênes et al. (2009) with those of Hack et al. (2005) and Zhang et al. (2015), I expect prenatal exposure to high ambient temperatures to reduce cooperative behavior. I analyze this relationship by fitting the following linear probability model (LPM):

(1) $$\begin{array}{l} \;Pr(Cooperation_{iyms}=1|Temperature_{ym},{\text{x}}_{iyms},{\text{z}}_{iyms})=\\ \alpha +\beta Temperature_{ym}+\gamma \prime {\text{x}}_{iyms}+\delta \prime {\text{z}}_{iyms}+\zeta_{m}+\eta_{s}+\varepsilon_{iyms} \end{array}$$

where *Cooperation*_*iyms*_ equals 1 if child *i* born in month *m* of year *y* and attending school *s* selects the cooperative option, *Temperature*_*ym*_ is the mean ambient temperature during the likely gestation of children born in month *m* of year *y*, **x**_*iyms*_ is a vector of individual child characteristics (female, age, age×female), **z**_*iyms*_ is a vector of caregiver characteristics (female, age, age×female, Acholi, years of education), ζ_*m*_ are month-of-birth fixed effects, η_*s*_ are school fixed effects, and ε_*iyms*_ is a stochastic error term. Standard errors are clustered at the level of running month of birth.

Estimating the model without controls, I find that exposure to high ambient temperature during gestation is negatively correlated with the child's probability of contribution to the public good. Parametrically, a 1°C increase in mean ambient temperature during gestation reduces the child's probability of contribution by 7.6% points (Table 3, column 1). At mean prevalence of 25.6%, this is equivalent to a 30% reduction in the likelihood of cooperation.

**Table 3:** High temperature decreases taste for cooperation.

	**Child cooperation**				
	**(1)**	**(2)**	**(3)**	**(4)**	**(5)**
Temperature	−0.076^**^	−0.134^**^	−0.140^**^	−0.138^*^	−0.204^***^
	(0.038)	(0.055)	(0.069)	(0.073)	(0.069)
Month of birth FE	N	Y	Y	Y	Y
Child characteristics	N	N	Y	Y	Y
Caregiver characteristics	N	N	N	Y	Y
School FE	N	N	N	N	Y
*N*	531	531	531	522	522
Area under ROC curve	0.556	0.612	0.618	0.624	0.756

*Notes: SE clustered at the level of running month of birth in parentheses*.

* *p < 0.10*,

** *p < 0.05*,

*** *p < 0.01*.

*Child characteristics: Female, Age (in months), Age × Female. Caregiver characteristics: Female, Age (in years), Age × Female, Acholi, Years of education*.

To account for non-temperature seasonal confounds and unobserved background characteristics potentially related to season of birth similar to those described by Buckles and Hungerman (2013) in the United States, I include calendar month fixed effects, which only increases the magnitude of the detected effect of temperature (Table 3, column 2). The relationship could potentially also be driven by other child characteristics. Prosocial preferences develop throughout childhood and adolescence, and become increasingly gender-dependent with approaching adulthood (Eisenberg et al., 2006). Controlling for age, gender and their interaction, however, does not change the interpretation of the result (Table 3, column 3), nor does controlling for caregiver characteristics and school fixed effects to account for family and peer demographics (Table 3, columns 4 and 5), both of which have been linked to children's prosocial behavior (Eisenberg et al., 2006).

#### Result 1

Exposure to abnormally high ambient temperature during gestation decreases later-life taste for cooperation. A 1°C (1 s.d.) increase in mean ambient temperature during gestation decreases the probability of cooperation in a public goods game by up to 20% points (10% points), leading to a 16% (8%) drop in total welfare.

The result holds when subjected to a battery of robustness checks. It remains practically unchanged when estimated by probit and logit models (see Table A.1 in the Supplementary Materials). It is not driven by outliers—excluding observations with high-leverage (the most extreme values of independent variables) and high-influence observations (observations whose deletion from the dataset would most change the magnitude of the estimated coefficients) does not significantly affect the result (see Table A.2, columns 2 and 3 in Supplementary Materials). Limiting the analysis to children born in the same area where they were interviewed also does not affect the result (see Table A.2, column 4 in Supplementary Materials). Including the mean air temperature during a 9-month period 1 year prior to the assumed period of gestation as a placebo treatment leaves the result unaffected, as does assuming other periods of gestation^15^, and using an alternative source of temperature data (see Table A.3 in Supplementary Materials).

**Figure 4:**
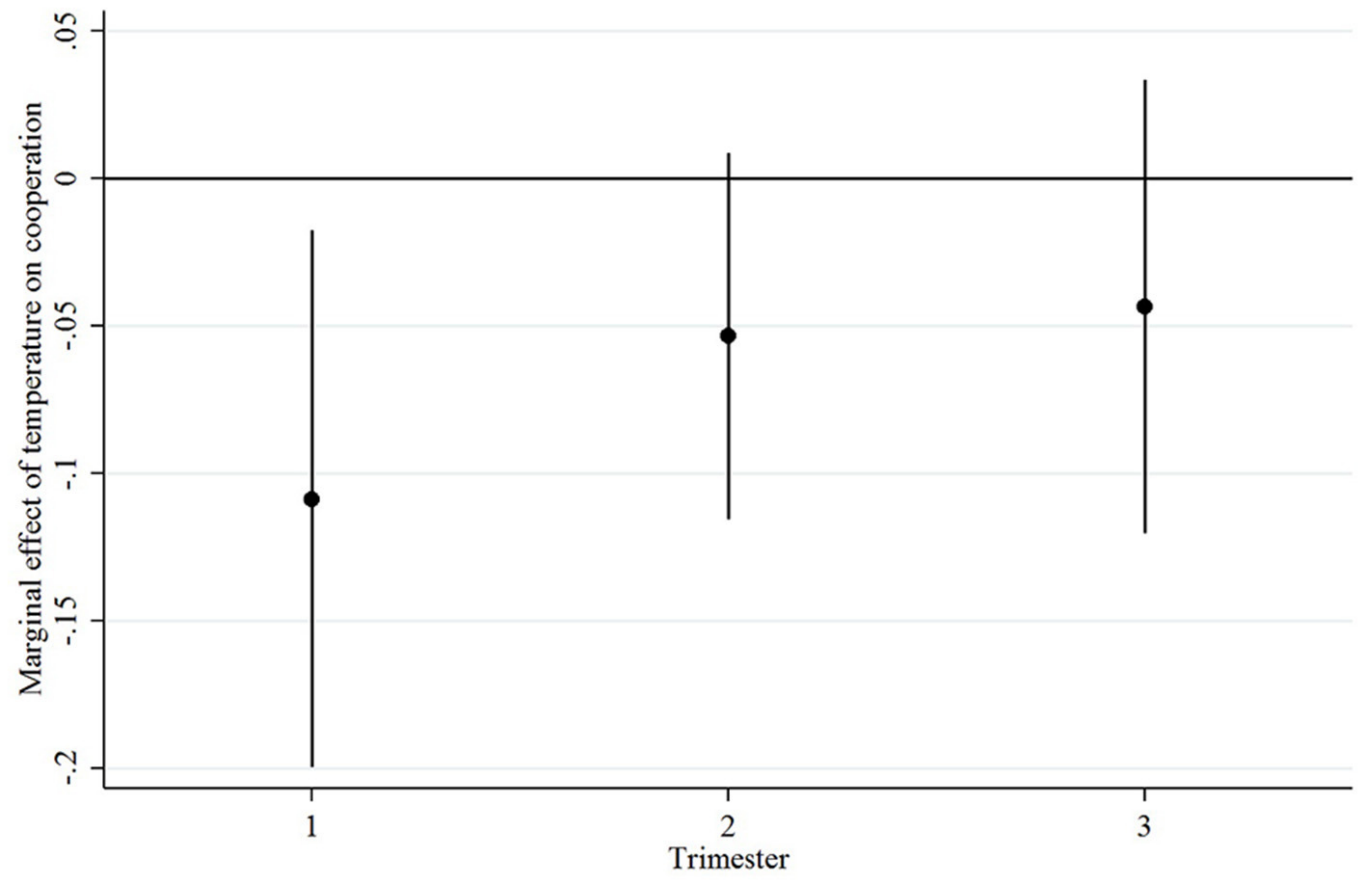
Temperature effects are strongest in the first trimester.

To better understand the main result, I estimate temperature effects on later life cooperation for each pregnancy trimester. This not only serves to better pinpoint the critical period of exposure, but also provides an indication of the potential mechanisms at play. The first trimester is crucial to brain development, and it is the time when epigenetic programming of the endocrine system takes place. The third trimester, when the fetus gains the most weight, is crucial for general health. It is clear from Figure 4, which shows the effects of mean ambient temperature in each trimester (and their 90% confidence intervals), that the result is driven mainly by exposure in the first trimester. The magnitude of the effect of temperature shocks in the first gestational trimester is about twice as large as those of temperature shocks in the second and third trimesters. The effect in the first trimester is also the only statistically significant one in my estimation (*p* = 0.05), but that could well be due to my underpowered estimation^16^. Additively, they make up the overall temperature effect throughout gestation. The fact that most of the effect seems to be concentrated in the first trimester suggests that the observed behavioral effects may be linked directly to altered brain development, changes in endocrine regulation, or both, rather than indirectly to general health.

### 3.2. Indirect temperature effects and other factors

Both in theory and in my data, temperature is strongly negatively correlated with precipitation, which in turn affects agricultural yields and—by extension—food prices. The combination of high temperatures and low precipitation during gestation could thus lead to malnutrition in infancy, whose negative consequences for the child's cognitive abilities can persist for years (Beckett et al., 2006). On the other hand, low precipitation levels decrease the likelihood of malaria contraction (Craig et al., 1999), and could thus also have a positive effect on later-life outcomes (Barreca, 2010).

Controlling for the environmental covariates and the indicators of early-life deprivation, I find that high precipitation during gestation decreases children's taste for cooperation, while high consumer prices increase it (Table 4, columns 1 and 3). This suggests that—at least in the context of Northern Uganda—the effects of precipitation during gestation on later-life prosocial preferences via exposure to malaria dominate those via agricultural yields, and that—unsurprisingly—the mean sampled household is likely to be a net food producer. Importantly, however, precipitation and consumer price effects do not wash away the effect of temperature itself (Table 4, columns 4 and 6).

**Table 4:** Other environmental factors and early life deprivation.

	**Child cooperation**					
	**(1)**	**(2)**	**(3)**	**(4)**	**(5)**	**(6)**
Temperature				−0.205^**^	−0.197^***^	−0.221^***^
				(0.087)	(0.070)	(0.082)
Precipitation	−0.079		−0.102^**^	−0.090^*^		−0.116^**^
	(0.048)		(0.050)	(0.050)		(0.050)
Consumer prices	0.020^**^		0.019^**^	0.004		0.001
	(0.009)		(0.009)	(0.011)		(0.010)
Height-for-age		0.023	0.026		0.020	0.024
		(0.021)	(0.021)		(0.020)	(0.020)
BMI-for-age		−0.039^*^	−0.048^**^		−0.044^*^	−0.048^**^
		(0.022)	(0.023)		(0.022)	(0.023)
IQ-for-age		0.004^**^	0.004^***^		0.004^***^	0.004^***^
		(0.001)	(0.001)		(0.001)	(0.001)
Child and caregiver	Y	Y	Y	Y	Y	Y
characteristics						
Month of birth	Y	Y	Y	Y	Y	Y
& School FE						
*N*	522	468	468	522	468	468
Area under ROC curve	0.751	0.782	0.787	0.760	0.792	0.798

*Notes: Probit marginal effects. SE clustered at the level of running month of birth in parentheses*.

* *p < 0.10*,

** *p < 0.05*,

*** *p < 0.01. Child characteristics: Female, Age (in months), Age × Female. Caregiver characteristics: Female, Age (in years), Age × Female, Acholi, Years of education*.

High cognitive abilities proxied by the age-adjusted IQ predict higher probability of contributing to the public good in accordance with Zhang et al. (2015). From Beckett et al. (2006), I would expect height-for-age and BMI-for-age—both markers of early-life nutritional deprivation—to also be positively correlated with child cooperation. Instead, I estimate their effects to be statistically insignificant and significantly negative respectively (see Table 4, columns 2 and 3). Their inclusion in the model does not however alter my main result (Table 4, columns 5 and 6).

There is increasingly conclusive evidence that high temperatures may trigger or intensify violent conflict (O'Loughlin et al., 2012; Hsiang et al., 2013). Pre- and post-natal exposure to conflict have in turn been found to influence social preferences: Conflict-induced prenatal stress reduces contributions to the public good in later life (Cecchi and Duchoslav, 2018), while post-natal exposure leads to more prosocial behavior within close networks (Voors et al., 2012; Bauer et al., 2014; Gilligan et al., 2014). Many of the children in my sample were born during a period of civil war in Northern Uganda. Using a sub-sample for which information on war exposure and prenatal stress is available,^17^ I find that prenatal stress (proxied by a z-score of the reverse 2D:4D ratio—a marker of prenatal stress) indeed reduces the taste for cooperation (Table 5, column 1). Unlike other studies (Voors et al., 2012; Bauer et al., 2014; Gilligan et al., 2014), I find no statistically significant relationship between postnatal conflict exposure and cooperation, though this could be due to the crudeness of my measure of conflict exposure (see section 2.2 for details). Importantly, the inclusion of these war-related controls does not wash away the effect of ambient temperature during gestation; it rather makes it stronger (Table 5, column 2).

**Table 5:** Conflict exposure.

	**Child cooperation**	
	**(1)**	**(2)**
Temperature		−0.250^**^
		(0.109)
Prenatal stress	−0.050^*^	−0.052^*^
	(0.027)	(0.027)
Postnatal conflict exposure	−0.013	−0.015
	(0.023)	(0.022)
Child characteristics	Y	Y
Month of birth and School FE	Y	Y
*N*	211	211
Area under ROC curve	0.869	0.883

Notes:

* *p < 0.1*,

** *p < 0.05*,

^***^ p < 0.01.

*SE clustered at the level of running month of birth in parentheses. Child characteristics: Female, Age, Age^2^, Age × Female, Age^2^× Female*.

The preferences of children may be influenced by those of their caregivers through both environmental and—when the two are blood related—genetic mechanisms (Dohmen et al., 2012). Controlling for caregiver preferences, I find that a child's social preferences are strongly correlated with the social preferences of their main caregiver, but not with the caregiver's risk preferences. Children are about 10% points more likely to contribute to the public good if their main caregiver contributes to to it as well in a separate game (Table 6, column 1). The effect of ambient temperature during gestation is however not affected by these controls (Table 6, column 2), and the results hold when analysis is restricted to caregivers who are biological parents of their children (Table 6, columns 3 and 4).

**Table 6:** Caregiver preferences.

	**Child cooperation**			
	**All caregivers**		**Biological parents only**	
	**(1)**	**(2)**	**(3)**	**(4)**
Temperature		−0.200^***^		−0.191^**^
		(0.070)		(0.075)
Caregiver cooperation	0.104^**^	0.103^**^	0.110^**^	0.113^**^
	(0.045)	(0.044)	(0.047)	(0.046)
Caregiver risk aversion	−0.028	−0.024	−0.004	0.002
	(0.037)	(0.037)	(0.042)	(0.041)
Child characteristics	Y	Y	Y	Y
Month of birth & School FE	Y	Y	Y	Y
*N*	522	522	462	462
Area under ROC curve	0.752	0.762	0.740	0.752

Notes: SE clustered at the level of running month of birth in parentheses. ^*^p < 0.10,

** *p < 0.05*,

*** *p < 0.01. Child characteristics: Female, Age (in months), Age × Female. Caregiver characteristics: Female, Age (in years), Age × Female, Acholi, Years of education*.

Finally, it is conceivable that different types of parents are more likely to conceive at times with different weather and climate patterns. If the different types of parents would also have different social preferences, such self-selection could bias my results. In my setting, much of any such bias should be absorbed by the month of birth fixed effects. To further verify that no self-selection bias is present, I regress a battery of caregiver characteristics on mean temperature during the child's gestation according to the following model:

(2) $$y_{iyms}=\alpha +\beta Temp_{ym}+\gamma '{\text{x}}_{iyms}+\delta_{m}+\zeta_{s}+\varepsilon_{iyms}$$

where *y*_*iyms*_ refers to one of the following characteristics of the caregiver of child *i* born in month *m* of year *y* in village *s*: gender, marital status, functional literacy, risk aversion, public goods game choice, age at birth of child, years of education, conflict exposure, wealth, and household size. All other notation is the same as above.

**Figure 5:**
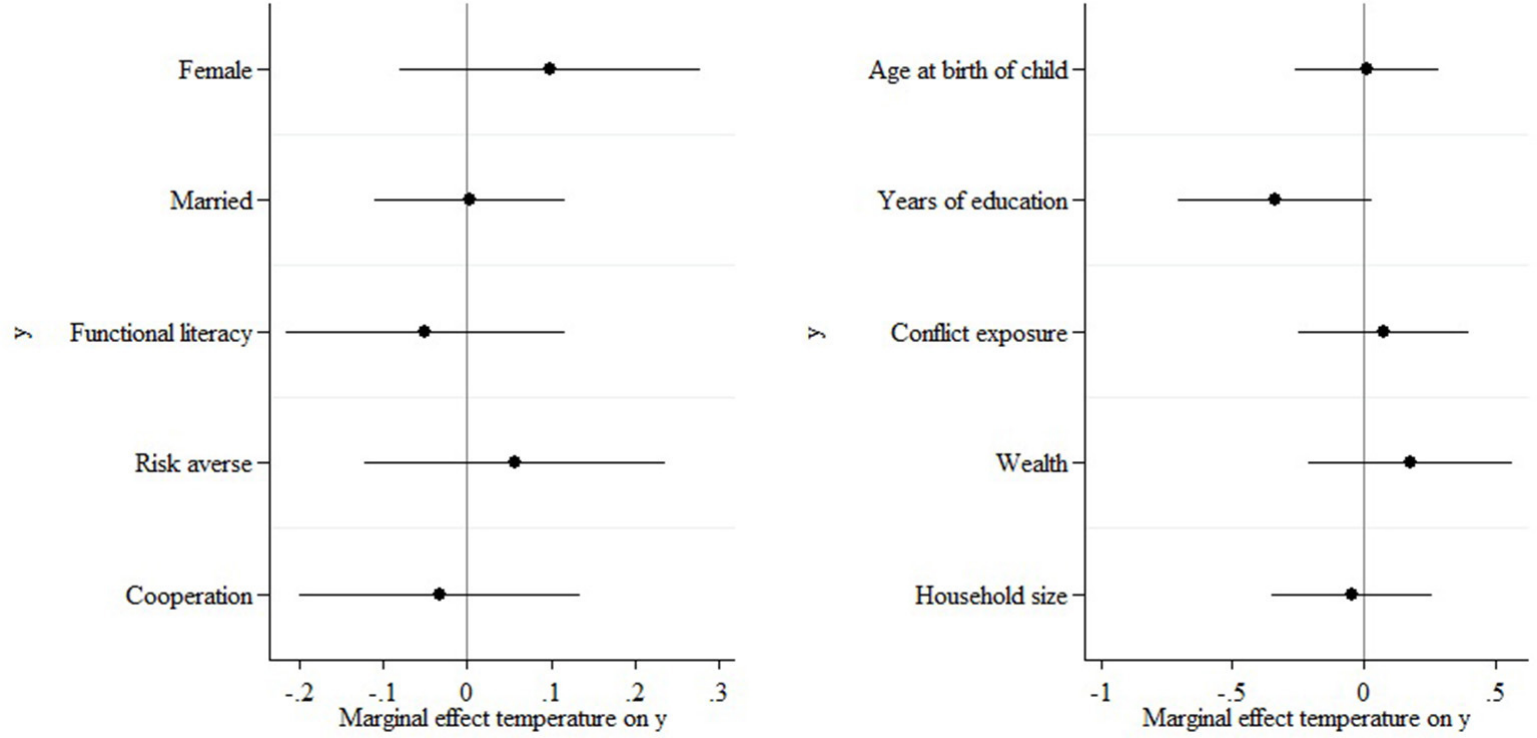
Caregiver self-selection does not drive the results.

If parents did not self-select into conceiving at the onset of a particularly hot (or cold) 9-months period based on these characteristics, the estimated β coefficients should be statistically insignificant. I summarize the estimated β coefficients and their 95% confidence intervals in Figure 5. As expected, none is statistically different from zero, indicating no detectable parent self-selection bias.

#### Result 2

The relationship between ambient temperature during gestation and cooperation is stable and robust to controlling for other environmental factors, early life deprivation markers, pre- and post-natal conflict exposure and caregiver preferences.

### 3.3. Long-term effects

To gauge the long-term effects of ambient temperature shocks during gestation on the taste for cooperation and to test the external validity of my main finding, I apply a similar analytical approach to a sample of adults from a different part of the country playing a different type of public goods game. I first fit the following OLS model:

(3) $$Contribution_{iyms}=\alpha +\beta Temperature_{ym}+\gamma '{\text{x}}_{iyms}+\delta_{m}+\zeta_{s}+\varepsilon_{iyms}$$

where *Contribution*_*iyms*_ represents the average amount of tokens contributed to the public good by participant *i* born in month *m* of year *y* and living in village *s*. *Temperature* has the same meaning as above. **x**_*iyms*_ is a vector of personal characteristics of participant *i* born in month *m* of year *y* and living in village *s*, which is comprised of *Age* (age in months), *Female* (a dummy equal to 1 if the participant is female), and their interaction. δ_*m*_ are month of year fixed effects, ζ_*s*_ village fixed effects, and ε_*iyms*_ is a stochastic error term. Standard errors are clustered at the levels of running month of birth and game group.

Estimating the model both with and without controls, I find a negative and statistically significant effect of ambient temperature during gestation on contribution to the public good, with every 1°C increase in temperature lowering contributions to the public good by nearly 0.5 token or some 13% (Table 7). The result is robust to outlier exclusion (see Table A.4 in the Supplementary Materials) as well as to a placebo test by older temperatures (see Table A.5 in the Supplementary Materials).

**Table 7:** Long-term effects.

	**Contribution**	
	**(1)**	**(2)**
Temperature	−0.341^*^	−0.468^**^
	(0.193)	(0.204)
Personal characteristics	N	Y
Month of birth & Village FE	N	Y
*N*	257	257
adj. *R*^2^	0.006	0.140

*Notes: SE clustered at the level of running month of birth and game group in parentheses*.

* *p < 0.10*,

** *p < 0.05*,

^***^p < 0.01. Personal characteristics: Female, Age, Age × Female.

#### Result 3

The negative effects of exposure to unusually high ambient temperature during gestation on later-life taste for cooperation last into adulthood. A 1°C (1 s.d.) increase in mean ambient temperature during gestation decreases contributions to the public good by about 13% (5%), thus decreasing total welfare by 6% (2%).

## 4. Discussion and conclusion

When Montesquieu (1748) wrote that excess heat makes people “slothful and dispirited,” he pointed out that the fact is often used as a justification for slavery. It is perhaps due to the negative connotations of this argument that few social scientists studied the effects of heat on human behavior until quite recently. With global climate change driving temperatures to historically unprecedented levels, this attitude has drastically shifted.

There is now some cross-country evidence suggesting that prevailing extreme temperatures negatively affect health outcomes (Wells and Cole, 2002), and hamper economic production (Burke et al., 2015). Looking exclusively at such cross-country studies, one could be tempted to conclude that it is absolute temperature that drives health and behavioral changes, and that geographical location largely predetermines health and economic outcomes. In such a world, children in tropical countries would be born underweight (Wells and Cole, 2002), suffer from the various negative consequences of poor birth outcomes (Black et al., 2007), and grow up in inefficient economies (Burke et al., 2015). In the context of this study, they would become less cooperative than their luckier counterparts from more temperate climates.

Within-country analyses, however, paint a more complex picture. Due to their longitudinal nature, they have to control for any trends and seasonal patterns not associated with temperature (typically by including time fixed effects in their models), effectively netting out seasonal and long-term temperature patterns as well. Their findings suggest that unexpected deviations from normal temperatures—rather than absolute temperatures—are responsible for observed health and behavioral changes (Dell et al., 2009; Deschênes et al., 2009; Hsiang et al., 2013). In the context of this paper, one would thus expect a person born in an unusually warm year in Northern Uganda to be less cooperative than their neighbor born in an unusually cold year. One would, however, not know whether they should be more or less cooperative than somebody born on the same day in North Holland, for example.

Relying on longitudinal data from two locations in Uganda, I follow Dell et al.'s (2009) recommendation to include time fixed effects in this paper. I find that exposure to higher than normal ambient temperatures during gestation reduces the probability that a child contributes to the public good. The estimated effect is large, and lasts into adulthood. It is most pronounced in the first gestational trimester, which is consistent with the hypothesis that the mechanism through which temperature shocks during gestation alter later-life behavior is linked directly to altered brain development, changes in endocrine regulation, or both, rather than indirectly to general health. Due to the reduced form of this study, I cannot unfortunately make any conclusive claims in this regard, and I leave the establishment of precise causal links to future research into the physiological mechanisms of intrauterine programming—a topic of vigorous scientific debate. I do, however, show a clear correlation between abnormally high ambient temperatures during gestation and reduced cooperation in later life. The relationship is robust to controlling for potential confounders including other environmental factors, markers of early-life deprivation, prenatal stress, postnatal conflict exposure and caregiver preferences, and is therefore unlikely to be of spurious nature.

Thus, people's willingness to cooperate—a prerequisite for much of economic production—may decline as the likelihood of extreme temperatures increases. The welfare implications of this are substantial in my stylized behavioral games. Their estimation in practice is, however, beyond the scope of this paper, and should instead be the focus of future research. Similarly, it will be important to study the extent to which adaptation to new climatic realities may mitigate the behavioral effects of higher temperatures. Until these questions are answered, at least the possibility of such effects should be taken into account when constructing the damage function of climate change and assessing the benefits of climate policies.

## Ethics statement

The study was carried out in accordance with the recommendations of the Social Sciences Ethics Committee at Wageningen University. All subjects or their caregivers gave written informed consent in accordance with the declaration of Helsinki. The protocol was approved by the Social Sciences Ethics Committee at Wageningen University.

## Author contributions

The author confirms being the sole contributor of this work and approved it for publication.

### Conflict of interest statement

The author declares that the research was conducted in the absence of any commercial or financial relationships that could be construed as a potential conflict of interest.

## Footnotes

^1^Additionally, preterm birth—another marker of adverse intrauterine conditions—predicts poor educational attainment (Bhutta et al., 2002). The 2D:4D ratio—a marker of prenatal stress—predicts cognitive abilities (Bosch-Domènech et al., 2014), risk preferences (Sapienza et al., 2009; Cronqvist et al., 2016), and prosocial preferences (Buser, 2012; Brañas-Garza et al., 2013; Galizzi and Nieboer, 2015; Cecchi and Duchoslav, 2018).

^2^Wells and Cole (2002) come to a different conclusion, providing between-population evidence that absolute—rather than relative—temperature shocks during gestation drive changes in birth-outcomes. Although they control for various confounding factors such as income and nutritional intake, these are aggregated at the national and yearly levels. Due to this design, the study cannot distinguish between regular seasonal variation in temperature and relative temperature shocks.

^3^See Appendix B in Supplementary Materials for an overview of variable definitions.

^4^The dataset produced by Harris et al. (2014) at the Climate Research Unit at the University of East Anglia is clearly more popular than that assembled by Willmott and Matsuura (2015) at the University of Delaware. While Willmott and Matsuura's dataset has been cited by 149 studies since its publication in 2009, the CRU dataset has been cited 751 times since its publication 5 years later according to Google Scholar. In 2015 alone, the CRU dataset was cited in 226 publications on Africa, compared to only 20 citations of Willmott and Matsuura (including citations of previous versions of their dataset). I find the preference for Harris et al. odd, given that their data is based on a much sparser set of weather stations than Willmott and Matsuura's. Willmott and Matsuura use measurements from between 3 and 19 (on average 8) weather stations within a 5° (about 555 km) radius of my main research site in Northern Uganda, and between 3 and 19 (with an average of 6) stations within a 5° radius from my secondary research site in Southern Uganda, depending on the month and year of measurement. Harris et al.'s use only 0 to 4 (on average 3) and 0 to 2 (on average 1) stations respectively in the same regions, and until 1941, the nearest weather station used in their dataset was Harare—21° (2,300 km) from my main research site and 17° (1,900 km) from my secondary research site. Willmott and Matsuura's data predict over 25% of the variation in actual temperature data from Entebbe, Uganda, while Harris et al.'s predict less than 7%. I therefore consider Willmott and Matsuura (2015) superior to Harris et al. (2014) in the East African context, despite the overwhelming popularity of the latter.

^5^Out of a total of 672, the caregivers of 141 pupils did not know their children's birth date. These children were excluded from my analysis.

^6^Contrarily to many public goods games in which participants can choose their preferred contribution level, I opted for a dichotomous choice, effectively reducing the game to a prisoner's dilemma: respondents could either cooperate or not. While this reduced my ability to pick up the nuances present in the experimental sample, I believe that it facilitated the decision making process, especially for the youngest.

^7^Based on WHO recommendations for treating outliers (de Onis et al., 2007), I truncate the anthropometric data at 6 standard deviations from the mean. This results in 15 and 14 dropped observations for height-for-age and BMI-for-age respectively.

^8^The lengths of the index and ring fingers were measured on the palmar surface of the right hand, from the midpoint of the palmar digital crease to the tip of the finger. The state of the art in measuring finger lengths is to use an office scanner to take a perfectly flat image of the palmar surface of the hand, and computer software to measure the exact lengths. Given the constraints due to the remoteness of the field location, I instead used clipboards and tape measures, allowing only for precision to the nearest 1 mm. This resulted in measurement error of ±33.3% at the mean of the estimates. A pilot in which 30 raters each separately measured the digit lengths of 35 individuals revealed comparable margins of error. While the precision of this measurement is still well below that obtained in laboratory settings (see Voracek et al., 2007), my measurements should be at least as accurate as those in other field studies which sometimes only report whether the index finger is longer, shorter, or of the same length as the ring finger (Buser, 2012).

^9^Any questions about shelling and bombardment are irrelevant in the Ugandan setting, and were therefore omitted from the questionnaire.

^10^Source: ACLED Version 5, 1997-2013 (Raleigh et al., 2010).

^11^Nine caregivers did not know their age, reducing the number of observations to 522.

^12^Three caregivers refused to complete the conflict exposure module of the survey, reducing the number of observations to 528. This reduction carries over to the measure of child postnatal conflict exposure, which is derived from that of their caregiver.

^13^Out of a total of 450 randomly selected household representatives, 193 either did not know their month and year of birth, or did not show up to play the public goods game. These people were excluded from my analysis.

^14^Withholding the information about the exact length of the game helps ensure that all rounds, including the final one, are played in a strategic way.

^15^In my analysis, I assume the period of gestation to correspond with the calendar month of the respondent's birth and the previous 8 months. To obtain the mean temperature during the gestational period of a respondent born in November, for example, I average the mean temperatures from March until November. As discussed in section 2.1, this is quite a simplification. A child born full term on the 1st of November would have gestated between February and October, while a child born 1 month prematurely on the 30th of November would have gestated between April and November. As a robustness check, I re-estimate model (1) using these two extremes as alternative individual regressors.

^16^Since I estimate effect sizes as beta coefficients in a multivariate regression in one sample, I cannot directly analyze the power of the estimation in the sense of the probability of detecting a difference in the proportions or means of two samples, nor the related minimal detectable effect given the sample size. To get a rough idea of the power of the estimation, I split the sample at the median value of *Temperature*, and consider the half with high values of *Temperature* as shocked and the rest as not shocked (effectively recoding *Temperature* as a binary variable). Assuming these to be a treatment and a control group in an experiment, and requiring power of 0.80 and significance of 0.05, I could only detect a difference between the cooperation rates in the two groups 1.7 times larger than the observed one (or 1.5 times larger if I could use the full sample of 672 observations). Allowing for full variation in *Temperature* and using additional controls in the multivariate regression setting should improve the power of the estimation, but still likely leaves it far below ideal. To be sure, this does not mean that the estimates which I find to be statistically significant are not so. Rather, it means that I cannot rule out with sufficient certainty that the coefficients which which seem to be statistically insignificant in my estimations are not in fact different from zero.

^17^Part of the children in this study were also interviewed in 2012, at which time I measured the lengths of their fingers to calculate the 2D:4D ratio. I made the same measurements for this study, but after explaining to the research assistants that the digit ratio is “usually around 1”, the frequency of precisely that value being reported increased dramatically. While I do not believe that this was a result of intentional misenumeration, it does constitute a heavy bias, forcing me to discard the 2014 2D:4D values. This reduced the available sample to those children interviewed in 2012.
