# Supplementary material for: Prenatal Temperature Shocks Reduce Cooperation: Evidence from Public Goods Games in Uganda
Source: Front Behav Neurosci. 2017 Dec 21;11:249. doi: 10.3389/fnbeh.2017.00249 (PMC5742612; doi:10.3389/fnbeh.2017.00249)

Prenatal Temperature Shocks Reduce Cooperation: Evidence from Public Goods Games in Uganda

—

Supplementary Materials

Jan Duchoslav

A Tables

Table A.1: The main result is robust to alternative estimators

	Child cooperation		
	LPM	Probit	Logit
	(1)	(2)	(3)
Temperature	-0.204***	-0.208***	-0.209***
	(0.069)	(0.067)	(0.069)
Child & caregiver characteristics	Y	Y	Y
Month of birth & School FE	Y	Y	Y
N	522	499	499
Area under ROC curve	0.756	0.742	0.745

Notes: Linear (1), probit (2) and logit (3) marginal effects. SE clustered at the level of the month (by year) of birth in parentheses. * $p < 0.10$, ** $p < 0.05$, *** $p < 0.01$. Child characteristics: Female, Age (in months), Age×Female. Caregiver characteristics: Female, Age (in years), Age×Female, Acholi, Years of education.

Table A.2: The main result is robust to outlier exclusion and spatial restrictions

	Child cooperation			
	All	Low leverage	Low inluence	Born in the same village
	(1)	(2)	(3)	(4)
Temperature	-0.204***	-0.231***	-0.202***	-0.192***
	(0.069)	(0.055)	(0.067)	(0.070)
Child & caregiver characteristics	Y	Y	Y	Y
Month of birth & School FE	Y	Y	Y	Y
N	522	435	493	494
Area under ROC curve	0.756	0.740	0.736	0.737

Notes: SE clustered at the level of running month of birth in parentheses. * $p < 0.10$, ** $p < 0.05$, *** $p < 0.01$. Child characteristics: Female, Age (in months), Age×Female. Caregiver characteristics: Female, Age (in years), Age×Female, Acholi, Years of education. Low leverage: $h_{ii} < \mu_h + \sigma_h$. Low influence: $|DFBETA| < 2/\sqrt{N}$.

Table A.3: The main result is robust to a placebo test, to alternative assumptions about gestational period, and to using alternative data

	Child cooperation				
	Main	Placebo	Long gestation	Short gestation	CRU data
	(1)	(2)	(3)	(4)	(5)
Temperature	-0.204***	-0.182**	-0.231***	-0.188***	-0.135***
	(0.069)	(0.071)	(0.069)	(0.068)	(0.039)
Temperature (previous year)		-0.097			
		(0.077)			
Child & caregiver characteristics	Y	Y	Y	Y	Y
Month of birth & School FE	Y	Y	Y	Y	Y
N	522	522	522	522	522
Area under ROC curve	0.756	0.756	0.759	0.755	0.760

Notes: SE clustered at the level of running month of birth in parentheses. * $p < 0.10$, ** $p < 0.05$, *** $p < 0.01$. Child characteristics: Female, Age (in months), Age×Female. Caregiver characteristics: Female, Age (in years), Age×Female, Acholi, Years os education. Model (1) repeats the main result. Model (2) includes the mean temperature in the nine-month period one year earlier than the assumed gestational period as a placebo treatment. In model (3), I assume that children were born on the 1st day of their month of birth, and that they gestated full 9 months. In model (4), I assume that they were born on the last day of their month of birth, and that they only gestated 8 months. Model (5) uses temperature data from **?** instead of **?** (see Section **??** for details).

Table A.4: The long-term result is robust to outlier exclusion

	Contribution		
	All	Low leverage	Low influence
	(1)	(2)	(3)
Temperature	-0.468**	-0.489**	-0.730***
	(0.204)	(0.243)	(0.207)
Personal characteristics	Y	Y	Y
Month of birth & School FE	Y	Y	Y
N	257	222	222
adj. R^2	0.340	0.346	0.447

Notes: SE clustered at the level of running month of birth and game group in parentheses. * $p < 0.10$, ** $p < 0.05$, *** $p < 0.01$. Personal characteristics: Female, Age (in months), Age×Female. Low leverage: $h_{ii} < \mu_h + \sigma_h$. Low influence: $|DFBETA| < 2/\sqrt{N}$.

Table A.5: The long-term result is robust to a placebo test

	Contribution	
	(1)	(2)
Temperature	-0.468**	-0.403**
	(0.204)	(0.203)
Temperature (previous year)		-0.228
		(0.267)
Child characteristics	Y	Y
Month of birth & School FE	Y	Y
N	257	257
adj. R^2	0.140	0.140

Notes: SE clustered at the level of running month of birth and game group in parentheses. * $p < 0.10$, ** $p < 0.05$, *** $p < 0.01$. Personal characteristics: Female, Age (in months), Age×Female.

B Detailed variable definitions

Temperature. Mean ambient air temperature in the month of the respondent's birth and the preceding 8 months (in °C). All values are taken from **?**—a monthly time series interpolated to a 0.5°×0.5° grid. To obtain the mean temperature for the children, I averaged the values from four gird cells (2.5°N-3.5°N, 32.5°E-33.5°E) covering the former Pader district. Values for the adults in my secondary sample come from a single grid cell (0.5°S-1.0°S, 30.0°E-30.5°E), which covers all the surveyed villages.

Cooperation. A dummy representing the respondent's choice in a one-shot dichotomous public goods game: "group card" or "private card". The cooperative choice "group card" takes value 1, the non-cooperative choice "private card" takes value 0.

Female. A dummy taking the value of 1 if the respondent is female, 0 otherwise.

Age. Respondent's age in months (unless specified otherwise).

Height-for-age. The child's height standardized for his age class (**?**).

BMI-for-age. The child's body-mass-index standardized for his age class (**?**).

IQ-for-age. The child's IQ, measured using standard Raven's matrices and standardized for his age class (in sample).

2D:4D. A child level measure of the relative length of the index finger of the right hand with respect to the ring finger (in cm).

Prenatal stress. A child level variable derived by standardizing the 2D:4D (z-score): $DigitRatio_i = -\frac{2D{:}4D_i - \overline{2D{:}4D}}{\sigma}$. The negative sign is added for ease of interpretation.

(Postnatal) conflict exposure. The child's postnatal conflict exposure is proxied by the conflict exposure of his primary caregiver multiplied by the fraction of violent conflict events that took place after the child's birth. The caregiver's conflict exposure index is derived from answers to 23 war-witnessing questions (see **?**).

Precipitation. Mean precipitation in the month of the respondent's birth and the preceding 8 months (in mm/day). All values are taken from **?**—a monthly time series interpolated to a 0.5°×0.5° grid. To obtain the mean temperature for the children, I averaged the values from four gird cells (2.5°N-3.5°N, 32.5°E-33.5°E) covering the former Pader district. Values for the adults in my secondary sample come from a single grid cell (0.5°S-1.0°S, 30.0°E-30.5°E), which covers all the surveyed villages.

Consumer prices. Mean linearly de-trended consumer price index in the month of the respondent's birth and the preceding 8 months (**?**).

Age at birth of child. Caregivers age at her last birthday before the birth of the child.

Christian. A dummy equal to 1 if the respondent is Christian by religion, 0 otherwise.

Acholi. A dummy equal to 1 if the respondent is ethnically Acholi, 0 otherwise.

Munyankole. A dummy equal to 1 if the respondent is ethnically Munyankole, 0 otherwise

Years of education. The number of years of education the respondent completed.

Married. A dummy equal to 1 if the respondent is married, 0 otherwise.

Functional literacy. A dummy equal to 0 if the respondent never reads a newspaper, 1 otherwise.

Household size. The number of household members permanently residing in the homestead.

Wealth. An index constructed following **?** by taking the principal factor of assets possessed by the respondent's household.

Risk aversion. A dummy equal to 0 if the respondent chose the least risky of several lottery options; 1 otherwise.

Contribution. The mean number of tokens contributed over 5 rounds of a standard public goods game.

C Public goods game cards

Figure A.1: Individual and group cards

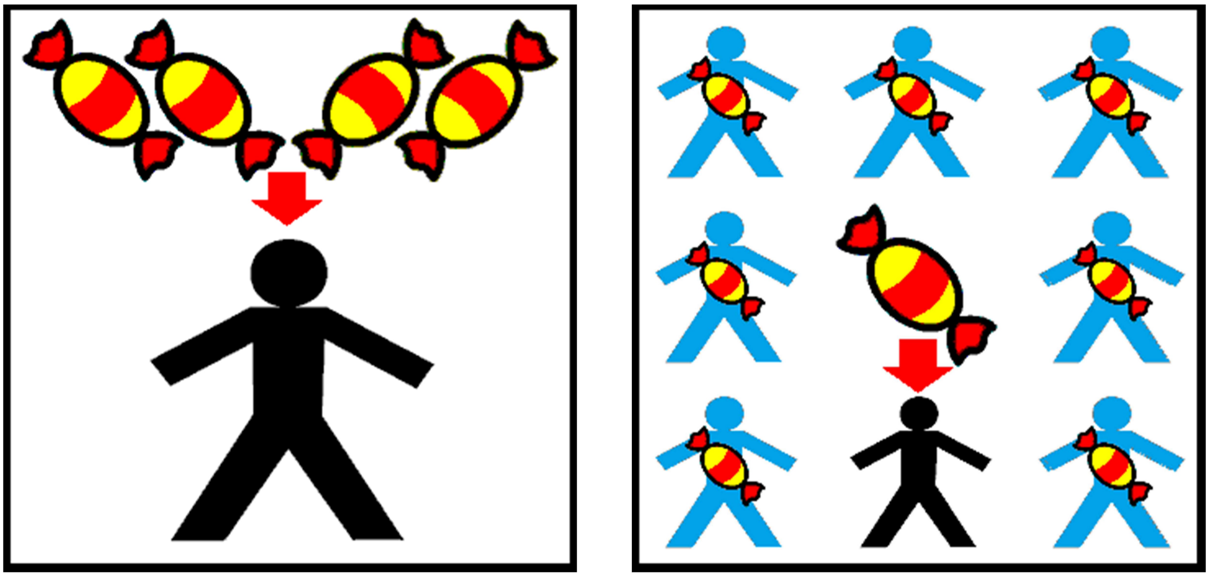

Supplement: Supplementary file 1 [file Table1.pdf]
